# *PLoS Computational Biology* Conference Postcards from ISMB/ECCB 2011

**DOI:** 10.1371/journal.pcbi.1002259

**Published:** 2011-11-17

**Authors:** Pedro Madrigal, Noa Sela, Simon M. Lin

**Affiliations:** 1 Institute of Plant Genetics, Polish Academy of Science, Poznań, Poland; 2 Ludwig Maximilians University, Munich, Germany; 3 Marshfield Clinic, Marshfield, Wisconsin, United States of America; University of California San Diego, United States of America

## Introduction

This July, *PLoS Computational Biology* invited attendees of ISMB/ECCB 2011 (http://www.iscb.org/ismbeccb2011) to send us short reports of conference highlights in the guise of PLoS Conference Postcards. Philip E. Bourne, Editor-in-Chief, selected three Postcards, which we received from Poland, Germany, and the United States of America. If the reports below capture your interest, you can find Postcards from past conferences in our recent collection: http://collections.plos.org/ploscompbiol/conferencepostcards.

## Alfonso Valencia on “Challenges for Bioinformatics in Personalized Cancer Medicine”

### Reported by Pedro Madrigal, Institute of Plant Genetics

What proteins can be found in a cell? How do protein complexes form, and why? How do gene families evolve, and what drives both gene duplications and epigenetic modifications? How is bioinformatics influencing personalized treatments of cancer cases? These four essential—and yet unanswered—questions were put forward to the ISMB 2011 audience by Professor Dr. Alfonso Valencia as the icebreaking launch pad of his keynote talk to challenge the community to develop a concerted effort in the field.

In the past few years, it has become evident that alternative splicing is one reason why human genomes can produce so much complexity with so few genes [1], with more than 50% of multi-exon human genes able to produce spliced mRNAs. One type of alternative splicing is characterized by clusters of internal exons being spliced in a mutually exclusive manner, but it constitutes a very rare case. It is known that most alternative splicing events produce isoforms very different than the *main* one, and “possibly isoforms we are not detecting are the ones important in oncogenic diseases”, Valencia pointed out, while indicating that for the vast majority of alternative isoforms there is still little evidence of their role as functional proteins. It has been suggested that, as a result of some disease events, potentially deleterious splice variants more or less dormant within the gene may be activated and highly expressed [2]. Valencia and colleagues have detected 204 genes with alternative splice variants, most of them subtly different from their constitutive counterparts. More information is available at the APPRIS web server (http://appris.bioinfo.cnio.es/), developed at the Spanish National Cancer Centre (CNIO).

How do proteins manage to distinguish the right binders (cognate interaction partners) from the wrong ones? To address this second question, Valencia reported a high-throughput docking experiment, showing that physical docking can often identify correct binders by predicting the interaction partners and the organization of the interaction surface using the distributions of the docking scores for over 1 billion of complex models generated [3]. Valencia's team has shown that it is possible to distinguish the structure of protein complexes by means of docking algorithms for 56 known interactors in their unbound form and a background of 922 non-redundant potential interactors. The formation of nonspecific “encounter complexes” helps to differentiate true binders by retaining many different conformations close to the final binding configuration. To achieve a comprehensive definition of protein function, Valencia showed the crucial role of protein interactions for the divergence generated during the evolution of protein families [4]. It is reflected on certain characteristic patterns of differentially conserved residues in protein subfamilies, known as “specificity determining positions”.

But, why do cancer cells accumulate structural variations? Is tumor progression analogous to species evolution? Is gene duplication a positively selected process, or is it an inevitable consequence of the mechanism of DNA replication? Both chromatin structure and DNA replication dynamics play a role in eukaryotic genomic evolution, and replication induces cellular stress, with exposed single-strand DNA leading to DNA damage. In the third part of the talk, Valencia put together DNA replication dynamics [5], [6], chromatin structure and gene age determination by phylostratification of evolutionary trees for each human gene [7]. Then we obtained a surprising result: old genes replicate earlier while newer genes replicate later in the cell cycle. Genes replicating later are found to be in heterochromatin-rich regions, and as a consequence of this process the specialization and diversification takes place in cell development. Valencia thus presented some beautiful examples of “how complicated things evolve”, as expressed by ISMB blogger Dr. Barbara Bryant (Constellation Pharmaceuticals), with whom I had the opportunity to discuss afterwards. It seems to be clear that determining replication-timing profiles may help to identify aberrations or alterations in replicating timing associated with disease [6]. The whole picture shows sort of “mechanistic process instead of selection driven”, stressed Valencia.

Subsequently, the talk went deep into the title topic, highlighting a recent review in the field [8]. Valencia outlined the following challenges in personalized cancer medicine: next-generation sequencing must evolve in technology and software; consequences of mutations in genes and proteins need to be unraveled; cancer gene mapping in functional pathways should make use of protein networks; and text and database mining have to be more effectively applied in drug design and pharmacogenetics. Today, patients' genomes are rarely consulted for diagnoses and treatment planning. Valencia remarked on the unique case of a pancreatic cancer patient whose tumor DNA was sequenced [9], and for whom “treatment was adjusted directly based on genome analyses”. The identification of the *PALB2* gene, previously associated with breast cancer predisposition as the second most commonly mutated gene for hereditary pancreatic cancer, allowed a better and rationally targeted personalized treatment provided by Manuel Hidalgo (CNIO) and colleagues [10], [11].

Last, but not least, Valencia underlined the contribution of Spain to the International Cancer Genome Consortium (ICGC) [12]. As a contributing member, the CLL Research Consortium will generate a comprehensive catalog of genetic alterations in 500 independent tumors of chronic lymphocytic leukemia (CLL). Results (from just first four cases) of whole-genome sequencing of CLL combined with clinical outcomes have identified clinically relevant mutations that contribute to the evolution of the disease [13].

The final take-home message was a call to stimulate the interchange of methods (software) and data (validated sets) within the scientific community, promoting in-harnessed collaborations across research groups. As Valencia said, “there is no gain by developing these systems in isolation or implementing only everyone's own software”.

Did Valencia achieve his purpose of challenging the audience? According to Bryant, “I was definitely interested right from the start. One of the things I liked about the talk was that he presented information I had not known about, and it really got me thinking”. If we consider the high number of questions in the discussion following the presentation—I counted nine in 11 minutes—the high impact it had on the attendees becomes evident. A good talk has the audience making guesses and I felt that Dr. Valencia did that well.

To sum up, recent developments in molecular biology aided by computing are paving the way in the era of genomics medicine, and new opportunities are emerging to detect genetic events leading to further progression of cancer. In my opinion, it will change the assumptions under which conventional treatments such as radiotherapy or chemotherapy are applied today to each patient, where the precise nature of genetic damage and the mutations involved are not yet well known. Thus, facing the up-to-date challenges expounded by Valencia may be considered as the next stepping stone to the utilization of personal genomics in forthcoming individualized cancer treatments.

## Milana Frenkel-Morgenstern on “Potential Functions of Proteins Encoded by Chimeric RNAs”

### Reported by Noa Sela, Ludwig Maximilians University

Many interesting lectures were given at the ISMB 2011 conference in Vienna. In my opinion, one of the outstanding sessions in the conference was the work dedicated to understanding the mysterious role and function of proteins encoded by chimeric transcripts, which was presented by Milana Frenkel-Morgenstern, a post-doctorate fellow in the CNIO in Madrid, Spain. Alternative splicing is thought to influence more than 70% of human genes and has a major contribution to both transcriptomic and protemic diversity. It has been shown to have a role in several genetic diseases as well as in cancer development. Chimeric transcripts may be generated by *trans*-splicing of pre-mRNAs or, alternatively, through gene fusion following translocations and rearrangements. Chimeric transcripts are of special interest since many of them have been shown to be associated with cancer. Nevertheless, very few chimeric transcripts, and especially their associated protein products, have been characterized. Their functional importance has remained mysterious and prompted the questions in the work presented by Dr. Frenkel-Morgenstern. The major aim of her work was to detect and functionally characterize the chimeric proteins products associated with genome-wide detection of chimeras by computational methods. Dr. Frenkel-Morgenstern explained that a significant proportion of the chimera transcripts were also shown to be present in normal cells; furthermore, many of the chimeras showed a tissue-specific expression pattern. Among all species analyzed, a substantial number of chimeras demonstrated a tendency for protein domain preservation, indicating constraints on protein product functionality of chimeric proteins. Another indication of functionality rises from enrichment of membrane proteins found within chimeras of humans, mice, and fruit flies. The most striking and important result of this research is indicated by the fact the 14% of chimeric proteins in humans may produce a dominant negative effect in cells. This finding indicates the importance of these transcripts' regulation in cells, albeit their potential rare abundance. It may also account for their association with pathogenesis and cancer.

By using the above genome-wide detection of chimeras and their functionality analysis, many specific events of special interest could be identified. For example, the chimera resulting from the fusion of the transcription repressor (Ctbp1) and transcription factor-3 (TCF3) produces a dominant negative protein that deactivates transcription. Another example showed the incorporation of signal peptide and transmembrane domain resulting from the fusion of solute carrier family 22 member 6 protein (Slc22a6) and thioredoxin domain-containing protein 12 (Txndc12).

I think that this talk raised an important discussion about the consequences of generation of chimeric proteins in cells. These chimera are likely to have substantially different functions than the original native proteins. This work indicates that it is feasible that these chimeras could have acquired specific functions and that they might exert dominant negative effects due to the absence of certain functional domains and therefore might compete with functional wild-type proteins.

Generally, I found that this talk was a good illustration of how experimental biology can benefit from computational approaches. The ISMB conference encourages the usage of advanced computational methods that resolve biological problems, which I believe was also exemplified by this talk. My personal feeling is that the work presented by Dr. Milana Frenkel-Morgenstern illustrates how important and valuable the use of computational methods is along with high-throughput screening for the analysis of protein functionality and characterization, and how they could contribute new hypotheses and insights for answering biological questions.

## Søren Brunak on “Integrating Phenotypic Data from Electronic Patient Records with Molecular Level Systems Biology”

### Reported by Simon M. Lin, Marshfield Clinic

Professor Brunak (Technical University of Denmark and University of Copenhagen) presented the first talk in the BioLINK special session at ISMB 2011 on how to utilize a systems biology approach to look at diseases phenotypes. The BioLINK session was organized by Christian Blaschke (Bioalma, Spain), Lynette Hirschman, (MITRE, United States), Hagit Shatkay (University of Delaware, United States), and Alfonso Valencia (Spanish National Cancer Research Centre, Spain).

With the BioLINK session focusing on data integration and interoperability across the computational, biological, and medical fields, Dr. Søren Brunak reported new gene–disease associations that have been discovered by integrating phenotype data with molecular data. In his talk, Dr. Brunak demonstrated how his group utilized electronic health records (EHRs) of Danish patients to extract patient-level phenotypic data. Unlike the United States' recent Medicare and Medicaid incentives, the Danish government launched their national strategy for EHRs much earlier, in the 1990s. Fortunately, there are still a few health care providers in the US, such as Marshfield Clinic, that have multiple decades of clinical data in the form of EHRs. These EHR datasets across the continents make it possible for future cross comparison and validation of the findings by Dr. Brunak's group.

A limitation indicated in Dr. Brunak's talk is that the connections between the molecular entities (for example, genes) to diseases are only at an aggregated level. In specific, the molecular data were text-mined from the OMIM database and other scientific literature. As such, patient-level variations, which are the crux of personalized medicine, were lost. As Dr. Brunak pointed out, molecular measurements from a biobank of patients can potentially solve this problem. The well-curated biobank with links to EHRs can be used to characterize the genotype-phenotype variation at the patient level. And several well-established biobanks in the US, such as BioVU at Vanderbilt University and the Personalized Medicine Research Project (PMRP) at Marshfield Clinic, can offer help.

EHRs remain a rather unexplored, but potentially rich, data source for most computational biologists. Dr. Brunak's avant-garde work represented the forefront of translational bioinformatics, which is defined as “the storage, retrieval, analysis, and dissemination of molecular and genomic information in a clinical setting”. Both the International Society of Computational Biology (ISCB) and American Medical Informatics Association (AMIA) are actively promoting translational bioinformatics.

**Figure 1:**
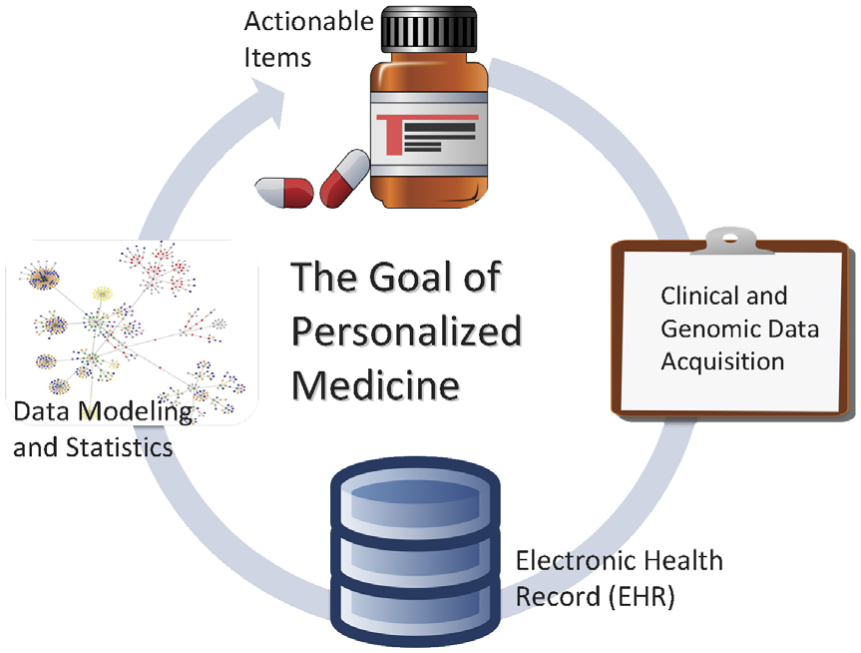
The goal of personalized medicine requires cross-fertilization between the disciplines of bioinformatics and medical informatics.

The disciplines of bioinformatics and medical informatics are closely related and they can be synergized to achieve the goal of personalized medicine (Figure 1). The attendees, speakers, and graduate training programs at the ISMB and AMIA annual meetings overlap at the grassroots level. From an organizational level, the cross-fertilization of bioinformatics and medical informatics has already borne fruit. For instance, the AMIA Summit on Translational Bioinformatics in 2009 was co-sponsored by ISCB. Many members of the two societies were cross-trained in both bioinformatics and medical informatics. Speaking from my own experience, I find my training in medical informatics gave me an edge working in bioinformatics, while the working experience in bioinformatics helped me explore further in medical informatics.

In summary, the convergence of bioinformatics and medical informatics can open new paths of exploration for personalized medicine. Dr. Brunak's talk was a good indication that more future joint activities by both ISBM and AMIA will benefit both current and future generations of biomedical informatics professionals.
